# Composite Materials Based on Biochar Obtained from Tomato Wastes and Fe_3_O_4_/MnO_2_ Used for Paracetamol Adsorption

**DOI:** 10.3390/ma18163914

**Published:** 2025-08-21

**Authors:** Adina Stegarescu, Ildiko Lung, Alin Cârdan, Mariana Bocșa, Alexandru Turza, Mihaela Diana Lazar, Monica Dan, Septimiu Tripon, Irina Kacso, Stelian Pintea, Ocsana Opriș, Maria-Loredana Soran

**Affiliations:** 1 National Institute for Research and Development of Isotopic and Molecular Technologies, 67-103 Donat, 400293 Cluj-Napoca, Romania; adina.stegarescu@itim-cj.ro (A.S.); ildiko.lung@itim-cj.ro (I.L.); alexandru.turza@itim-cj.ro (A.T.); diana.lazar@itim-cj.ro (M.D.L.); monica.dan@itim-cj.ro (M.D.); septimiu.tripon@itim-cj.ro (S.T.); irina.kacso@itim-cj.ro (I.K.); stelian.pintea@itim-cj.ro (S.P.); loredana.soran@itim-cj.ro (M.-L.S.); 2 Faculty of Environmental Sciences and Engineering, Babeș-Bolyai University, 30 Fântânele, 400294 Cluj-Napoca, Romania; cardanalin1@yahoo.com; 3 Faculty of Food Engineering, Tourism and Environmental Protection, Aurel Vlaicu University, 310130 Arad, Romania; marianabocsa@yahoo.co.uk

**Keywords:** tomato wastes, biochar, metal oxides, composites, paracetamol, adsorption

## Abstract

The pharmaceutical contamination of water, especially by widely used drugs, presents important environmental and health concerns due to the inefficiency of conventional treatment methods. The present study proposes a sustainable solution using biochar (Bch) obtained from tomato waste, functionalized with Fe_3_O_4_ and MnO_2_ nanoparticles, for the removal of paracetamol from aqueous solutions. The composite materials were synthesized, characterized, and evaluated under varying conditions, including pH, temperature, contact time, initial drug concentration, and adsorbent dose. The materials exhibited porous structures with wide pore size distributions. Optimal removal efficiency was achieved for 30 mg L^−1^ paracetamol concentration, pH 2, 25 °C, 0.3 g L^−1^ adsorbent dose, and 20 min contact time. The Freundlich isotherm provided the best fit for the adsorption data. Kinetic studies revealed that the pseudo-second-order model best described the adsorption process. Thermodynamic parameters indicated that the process was spontaneous, feasible, and exothermic. Compared with similar materials derived from agricultural waste, the tomato waste-based composites demonstrated competitive adsorption capacities. These findings suggest that Bch-HCl/MnO_2_ and Bch-HCl/Fe_3_O_4_/MnO_2_ are promising, cost-effective adsorbents for mitigating pharmaceutical pollutants in wastewater.

## 1. Introduction

A high number of individuals, particularly in Africa and Asia, face restricted access to safe drinking water, largely because of contamination by harmful chemicals. As the global population continues to rise, it is assumed that this issue will become even worse. Different pollutants, such as pharmaceuticals, dyes, and pesticides, enter the water sources, especially because of human activities [1,2]. Pharmaceutical compounds have become common contaminants of the global water cycle, deriving from diverse sources and persisting through the known conventional treatment processes. Numerous studies have documented the global presence of these types of pollutants [3,4,5].

Urban wastewater (municipal and industrial), hospital and pharmaceutical factory effluents, agricultural runoff (veterinary drugs, pesticides), and improper disposal (unused medication, landfill leachate) all contribute to the entry of pharmaceuticals into surface and groundwater [6,7]. Humans and livestock excrete unmetabolized pharmaceuticals and different metabolites into sewage, while hospitals and clinics discharge a significant amount of waste. For example, analgesics such as aspirin, naproxen, ibuprofen, and paracetamol (acetaminophen), which are widely used for pain relief, are frequently detected in rivers, lakes, and even treated wastewater effluent [8]. Once they have reached the environment, pharmaceuticals can move through waterways, infiltrate aquifers, and bioaccumulate in organisms, posing ecological risks to aquatic life and human health risks [9,10,11].

So far, studies have reported trace levels (ng–µg L^−1^) of different drugs in drinking water sources [10]. Paracetamol, the selected pollutant for this study, is a widely used medication prescribed for pain relief and is among the most consumed drugs globally. Consequently, it often enters aquatic environments through wastewater discharged from pharmaceutical facilities and hospitals [12,13]. Globally, paracetamol concentrations in wastewater can reach significant levels, ranging from 0.1 to 3.9 × 10^7^ ng L^−1^ [13,14]. Research has shown that paracetamol concentrations can reach up to 6 μg L^−1^ in effluents from European sewage treatment plants, up to 10 μg L^−1^ in natural waters (USA), and exceed 65 μg L^−1^ in the Tyne River (UK). In raw wastewater, paracetamol has been detected at a median concentration of 48 ± 75 μg L^−1^. However, in untreated hospital effluents and certain wastewater discharges, concentrations exceeded 150 μg L^−1^ [15]. The main issue with paracetamol lies in its metabolite, N-acetylimidoquinone, which is hepatotoxic and recognized for causing DNA damage. This byproduct is regarded as toxic, carcinogenic, and mutagenic [16,17,18]. In addition, paracetamol can negatively impact aquatic ecosystems by disrupting the metabolism of aquatic organisms and altering their antioxidant defense mechanisms [19,20].

Despite this global concern, traditional wastewater treatment plants (WWTPs) are not intended to eliminate these types of contaminants. Conventional treatment trains, primary settling, biological degradation, and filtration efficiently eliminate bulk organic load and nutrients but only partially remove pharmaceuticals [21]. As a result, only a small fraction of the pharmaceutical load is degraded or adsorbed during the treatment process, and most drugs (especially the persistent ones) pass further into the effluent. For example, paracetamol is often only partially degraded biologically and can persist through treatment [8]. The absence of regulatory requirements for emerging contaminants further extends this problem. Consequently, treated effluent often carries pharmaceuticals into receiving waters [8]. Advanced processes (advanced oxidation, membrane filtration, activated carbon) can improve removal but require high energy and cost. Overall, this highlights the need for the development of effective remediation strategies. Among the various methods used to eliminate these substances from water, adsorption stands out because of its excellent efficiency, affordability, and user-friendly operation [22,23]. In this context, affordable and eco-friendly materials are more suitable for the further and final treatment of wastewater containing pharmaceuticals.

Biochar, a carbon-rich solid obtained by thermochemical conversion of different biomass, has become a promising material for environmental remediation. In general, all organic precursors can be converted into biochar. Among the biomasses from which biochar was obtained are lignocellulosic materials (wood, crop residues, hulls), agricultural and animal wastes (straw, husks, manure), sewage sludge, and even invasive plant biomass [24,25,26]. Production methods include slow or fast pyrolysis, gasification, lower-temperature pyrolysis, and hydrothermal carbonization. Parameters such as temperature, residence time, and atmosphere are tuned to tailor biochar properties [27]. Using lower-temperature pyrolysis (300–500 °C) results in biochar rich in functional groups, whereas biochar obtained using higher temperatures can have a higher carbon content and surface area. Therefore, biochar production valorizes different residues and provides a sustainable route to obtaining low-cost adsorbents [8]. Generally, biochar has an extensive surface area and porous structure, favoring the sorption of organic compounds [8,28,29,30,31]. Compared to synthetic adsorbents, biochar is advantageous due to its low production cost, being environmentally friendly, and feasibility at a large scale [8].

Loading biochar with metal oxides is an effective modification strategy. Materials based on biochar–metal oxide often exhibit new synergistic properties that are not present in either component alone. Combinations with iron oxide (Fe_3_O_4_, magnetite) and manganese oxide (MnO_2_) have been studied intensively. These modifications make magnetic separation, pose catalytic activity, or additional adsorption functionalities [27,32].

Decorating biochar with magnetite nanoparticles confers magnetic properties, allowing the easy recovery of the adsorbent by magnetic separation [10]. MnO_2_ acts as a strong oxidant. The δ-MnO_2_ (birnessite) form can directly oxidize phenolic pharmaceuticals without any added oxidant. Paracetamol was efficiently oxidized by δ-MnO_2_, accompanied by concomitant production of Mn^2+^. There was an increase in the reaction speed at higher MnO_2_ doses and temperature but a decrease at higher pH values [33]. The transformation pathway involved the ring-opening and polymerization of the drug molecule. This suggests that MnO_2_-modified biochars can degrade pharmaceuticals via direct oxidation. In general, metal oxide modification alters biochar surface chemistry and reactivity. The dispersion of Fe_3_O_4_ or MnO_2_ in the carbon matrix can introduce positive charge sites (improving anion affinity), create new active sites for catalysis, and improve electron transfer properties [27]. The outcome is a versatile hybrid adsorbent/catalyst. It can first adsorb pharmaceuticals from water, concentrating them on the biochar surface, and then degrade them via metal-catalyzed oxidation. This dual action can lead to more complete removal than adsorption or oxidation alone.

From a technological perspective, developing affordable and effective treatment methods is very important. Biochar-based solutions directly address sustainability. The waste biomass is recycled and reduces carbon emissions relative to some conventional adsorbents [8]. They offer modularity (easy to integrate into existing plants or rural systems) and the potential for resource recovery (biochar can be regenerated or even reused for soil amendment after pollutant removal). Overall, metal-oxide-modified biochar represents a promising tool in water treatment. It exemplifies circular economy principles and could enable decentralized or point-of-use treatment systems. Future innovation will likely involve optimizing biochar architecture at the nanoscale, combining multiple activation methods, and integrating sensing/monitoring for smart treatment.

In this paper, the originality is given by the development of a novel adsorbent material based on biochar derived from tomato waste, which was further functionalized with Fe_3_O_4_ and MnO_2_ nanoparticles. The synthesized materials were characterized by different techniques. Then, the effect of different parameters on the adsorption property of the obtained materials was evaluated. Furthermore, the dual-functionalization with Fe_3_O_4_ and MnO_2_ enhances the adsorptive properties for paracetamol removal, a pharmaceutical pollutant of growing environmental concern. Additionally, converting waste materials that are typically discarded into useful resources supports efforts to mitigate environmental concerns. This strategy contributes to the circular economy, minimizes pollutant emissions, and enhances resource and energy efficiency [34,35]. To our knowledge, this specific combination of raw material, functionalization, and application has not been previously reported, resulting in the innovative point of our research.

## 2. Materials and Methods

### 2.1. Material Synthesis

#### 2.1.1. Preparation of Bch-HCl

**Figure 1:**
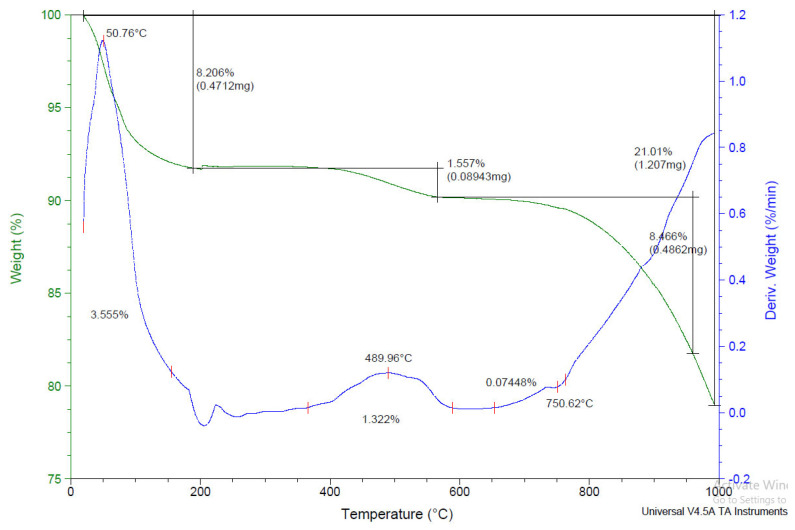
Mass loss (TGA) and derivative mass loss (DTG) during the pyrolysis of tomato processing waste.

The tomato wastes (skin), previously dried in the oven (Memmert GmbH + Co. KG, Schwabach, Germany) at 90 °C, were ground and then dried again in the oven at 130 °C for 48 h. In the second stage, it was calcined at 550 °C, in a tubular furnace (made at INCDTIM, Cluj-Napoca, Romania), in an Ar atmosphere, followed by slow cooling under an Ar flow. The conditions for the heat treatment were chosen following thermogravimetric (TGA) measurements (TA Instruments, New Castle, DE, USA). The TGA analysis was performed in the SDT Q 600 instrument (TA Instruments, New Castle, DE, USA) in an Ar atmosphere (100 mL/min) till 1000 °C with a heating rate of 10 °C/min. In the recorded spectra Figure 1, we observed a mass loss at 50.76 °C (8.2%), which accounts for most of the biomass mass loss, followed by a second, slower mass loss at 489.96 °C (1.55%). By combining the literature survey with the results obtained from TGA measurements, we have concluded that the optimal temperature for treating our material is 550 °C.

The biochar obtained in the previous step was mixed with a 10 M HCl solution for 30 min in an ultrasonic bath. The obtained suspension was washed with double-distilled water to neutral pH and dried overnight in an oven at 65 °C. Through this method, the activation of the biochar surface and the increase in the specific surface were attempted. In the subsequent step, the biochar was subjected to acid treatment by mixing it with 10 M HCl in an ultrasonic bath for 30 min. The resulting suspension was thoroughly washed with double-distilled water until a neutral pH was achieved and then dried at 65 °C overnight. This process was intended to activate the surface of the biochar and increase its specific surface area.

#### 2.1.2. Bch-HCl/MnO_2_ Synthesis

**Scheme 1:**
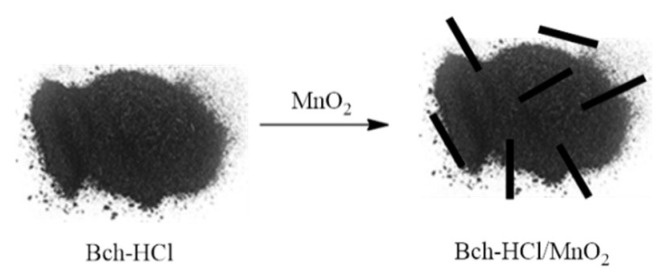
General scheme for obtaining the Bch-HCl/MnO_2_ composite.

Bch-HCl was suspended in double-distilled water and agitated on a shaker for 30 min. At the same time, MnO_2_ (prepared in the laboratory according to Lung et al. [36]) was mixed with double-distilled water until fully dissolved, in a mass ratio of MnO_2_ : Bch-HCl 5 : 1 *w*/*w* on a shaker, for 30 min. The MnO_2_ solution was introduced to the Bch-HCl dispersion, and shaking on the shaker was continued for 6 h at 400 rpm. The precipitate was recovered by centrifugation and subjected to three sequential washes with double-distilled water and then three times with ethanol. It was dried in an oven at 60 °C for 12 h (Scheme 1).

#### 2.1.3. Bch-HCl/Fe_3_O_4_/MnO_2_ Synthesis

**Scheme 2:**
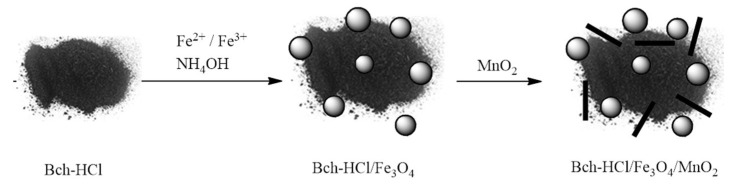
General scheme for obtaining the Bch-HCl/Fe_3_O_4_/MnO_2_ composite.

The biochar activated with HCl in the previous step was dispersed in water and ultrasonicated for 20 min, after which the suspension was transferred to the magnetic stirring plate and the solution was heated to 60 °C, under an inert atmosphere, for 30 min. Subsequently, FeCl_3_ × 6H_2_O was introduced, and the mixture was stirred for an additional 30 min. This was followed by the addition of FeSO_4_ × H_2_O, and stirring was continued at 60 °C for another 30 min. Afterward, 5.70 mL of 6% NH_3_ solution was added, and the reaction mixture was stirred further for 2 h under an inert atmosphere. Finally, the sample was washed using water until a neutral pH was reached and then left to dry in an oven at 65 °C overnight. At the same time, MnO_2_ (prepared in the laboratory according to Lung et al. [36]) was dissolved in double-distilled water, in a mass ratio of MnO_2_ : Bch-HCl 5 : 1 w/w on a shaker (IKA-Werke GmbH & Co. KG, Staufen, Germany), for 30 min. The MnO_2_ solution was mixed with the dispersed Bch-HCl/Fe_3_O_4_ and shaken for 6 h at 400 rpm. The precipitate was recovered by centrifugation and washed 3 times with double-distilled water and 3 times with ethanol, respectively. The resulting material was oven-dried at 60 °C for 12 h. (Scheme 2).

### 2.2. Materials Characterization

Three samples in suspension were brought to the Hitachi HD 2700 STEM electron microscope (Tokyo, Japan) for examination. From these samples, 6 µL for each sample was deposited on electrolytic grids with a diameter of 3 mm, mesh 300. The excess suspension from the grid was removed, leaving nanoparticles attached to the carbon film on the electrolytic grid. The samples were examined at 200 kV, obtaining alternative images both in scanning mode (SEM) and in transmission mode (TEM); this helped to achieve a better analysis by comparing the surface and the ultrastructure. Images were obtained at different magnifications between 25,000 and 200,000.

An EDX (energy-dispersive X-ray spectroscopy) analysis (Oxford Instruments, Tokyo, Japan) was also performed, with AZtec Oxford Instruments (software: Aztec version 3.3), the microscope being equipped with two detectors of this type.

The specific surface area (St) and pore size (Dp) were determined from N_2_ adsorption–desorption isotherms recorded at liquid nitrogen temperature (−196 °C) using a Bel-Sorp MaxX instrument (Microtrac BEL Corporation, Tokyo, Japan). Prior to analysis, the samples were degassed under vacuum at 200 °C for 4 h. The specific surface area was calculated by applying the Brunauer–Emmett–Teller (BET) method within the pressure range of 0.05 to 0.25 (p/p_0_). Pore size distribution was obtained by utilizing the Barrett–Joyner–Halenda (BJH) model on the desorption branch of the isotherm.

Fourier transform infrared (FTIR) spectra were recorded using a JASCO 6100 FTIR spectrometer (JASCO International Co., Ltd., Tokyo, Japan) over the range of 4000 to 400 cm^−1^ with a resolution of 4 cm^−1^, employing the KBr pellet method. Approximately 300 mg of anhydrous KBr was used to disperse each sample, which was then ground thoroughly in an agate mortar with a pestle. The resulting mixture was compressed into pellets using an evacuated die. Spectral acquisition and analysis were carried out with Jasco Spectra Manager version 2 software. The stability of the composites in acidic (pH 2) and alkaline (pH 10) environments was evaluated by FTIR spectroscopy following the procedure described by Lung et al. [36].

X-ray diffraction (XRD) patterns were collected at ambient temperature using a Rigaku SmartLab multipurpose diffractometer (Rigaku, Tokyo, Japan) equipped with Cu Kα_1_ radiation (λ = 1.54056 Å) generated by a 9 kW rotating anode. Data acquisition was managed through the SmartLab Guidance software (version 2.1.0.0, Rigaku, Tokyo, Japan). Measurements were conducted over a 2θ range of 7° to 85°, with a step size of 0.01°. The point of zero charge (pH_pzc_) was determined using the pH drift method as outlined by Lung et al. [36].

### 2.3. Adsorption Experiments

Paracetamol adsorption onto the prepared composites was conducted under static conditions, with variation in parameters including the initial solution pH (2–9), adsorbent dose (0.1–1 g L^−1^), contact time (5–30 min), temperature (20–40 °C), and initial drug concentration (30–50 mg L^−1^). For each experiment, 5 mL of paracetamol of a certain concentration and the corresponding amount of adsorbent were mixed in a 5 mL Berzelius beaker using a magnetic stirrer at 300 rpm. The experiments were conducted at various pH values and different temperatures. The initial pH of the paracetamol solution was adjusted with 0.5 N HCl or 5% NH_4_OH. After mixing for a certain time, the two phases were separated by filtration (Bch-HCl/MnO_2_) and by means of an external magnet (Bch-HCl/Fe_3_O_4_/MnO_2_). Following the separation of the supernatant, the concentration of residual pollutant was quantified by high-performance liquid chromatography (HPLC). Paracetamol analysis was carried out using a Shimadzu LC2010 HPLC system (Shimadzu, Tokyo, Japan) equipped with a photodiode array detector (PAD). Separation was achieved on a LiChrosorb RP-18 column (250 × 4 mm, 5 μm) maintained at 40 °C. Isocratic elution was performed using a mobile phase composed of acetonitrile and ultrapure water with 0.1% formic acid (65:35, *v*/*v*). The flow rate of the mobile phase was set to 1 mL min^−1^, with a column equilibration time of 5 min. An injection volume of 10 μL was used for each analysis. The removal efficiency (η, %) of paracetamol from the aqueous solution and the amount of paracetamol adsorbed onto the surface of the prepared composites, qt (mg g^−1^) were calculated using Equations (1) and (2), respectively, as follows:

(1) $$\eta \left(\%\right)=\frac{\left(C_{0}-C_{t}\right)\cdot 100}{C_{0}}$$

(2) $$q_{t}=\frac{\left(C_{0}-C_{t}\right)\cdot V}{m}$$

Co and Ct (mg L^−^^1^) denote the initial paracetamol concentration and the concentration at time t, respectively; V (L) refers to the volume of the paracetamol solution, while m (g) indicates the mass of the adsorbent applied.

### 2.4. Composites Reusability

To evaluate the regeneration capacity of the prepared composites, desorption experiments were performed using 0.1 M HCl ca regeneration agents. Desorptions were performed by suspending the 5 mg of composite loaded with paracetamol in 10 mL of 0.1 M HCl. The mixtures were stirred at 300 rpm for 20 min at room temperature, and finally, after separation of the composite, the concentration of paracetamol was determined by HPLC.

Five cycles of adsorption–desorption experiments were performed to determine the reusability of the prepared composites.

## 3. Results and Discussion

### 3.1. Materials Characterization

#### 3.1.1. Morphological Characterization

**Figure 2:**
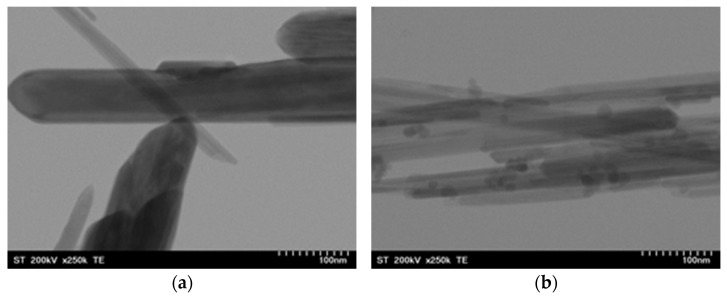
Representative TEM images for the investigated composites: Bch-HCl/MnO_2_ (**a**) and Bch-HCl/Fe_3_O_4_/MnO_2_ (**b**).

Shown in Figure 2 are the TEM images of the composites obtained. In general, the materials are not agglomerated, suggesting that these provide a large reactive surface area. For the Bch-HCl/MnO_2_ composite, it can be observed that the Bch-HCl has a spherical appearance, while for the MnO_2_ particles, their shape is comparable to that of “needles” due to the sharp morphology of the needle-like terminal ends. A variation in the size of the MnO_2_ needles and their fusion was observed. In the case of the Bch-HCl/Fe_3_O_4_/MnO_2_ composite, additional EDS tests could indicate the presence of spherical Fe_3_O_4_ nanoparticles as well as biochar nanoparticles. If agglomerations occur, they are due to the magnetic characteristics of the Bch-HCl/Fe_3_O_4_/MnO_2_ nanocomposite and are explained by the presence of magnetite.

**Figure 3:**
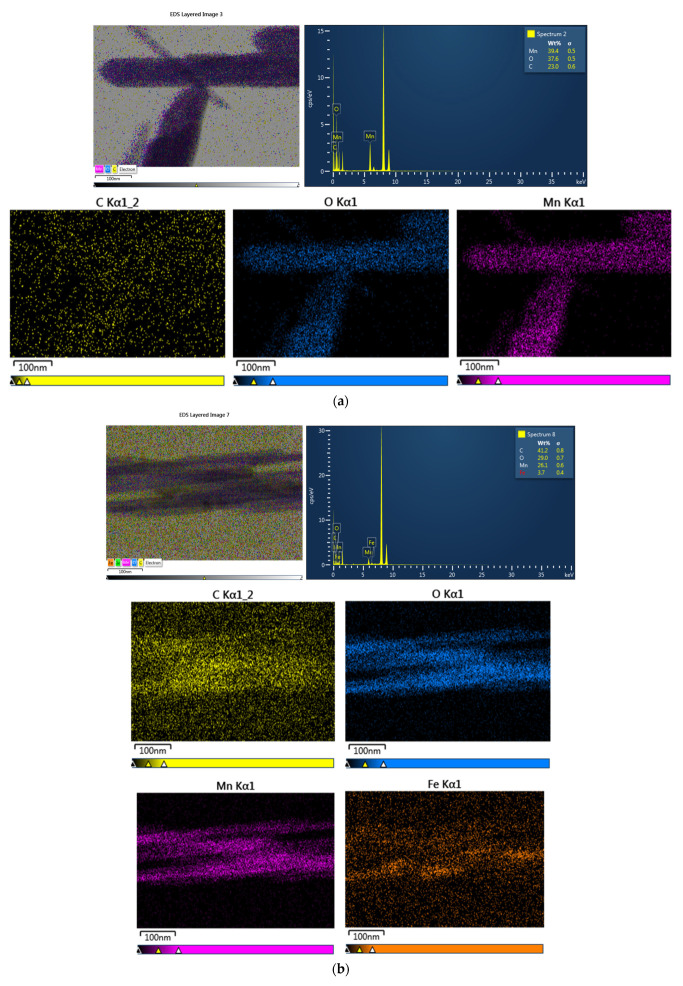
(**a**) The EDS images and the element distribution maps for Bch-HCl/MnO_2_. (**b**) The EDS images and the element distribution maps for Bch-HCl/Fe_3_O_4_/MnO_2_.

Figure 3a,b presents the EDS measurement results along with elemental distribution maps for the synthesized samples. Elemental analysis by EDX in the highlighted regions confirms the presence of all constituent phases. A uniform elemental distribution is observed across the selected surface of the examined particles. Carbon is detected as well, primarily originating from the carbon layer within the network and partially from the medium used to disperse the materials prior to analysis. 

#### 3.1.2. Surface Area and Porosity Analysis

**Figure 4:**
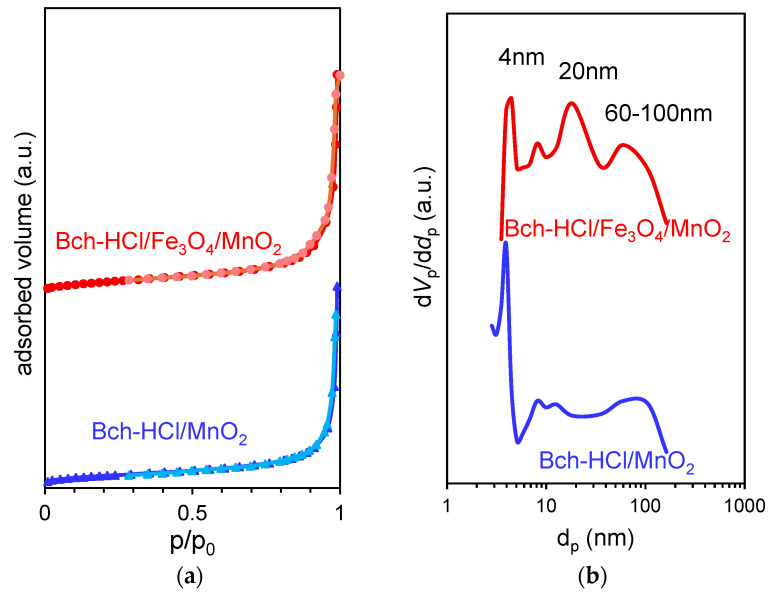
(**a**) Nitrogen adsorption and desorption isotherms and (**b**) pore size distribution of the prepared samples.

For both samples, the nitrogen isotherms of adsorption and desorption (Figure 4a) are of type IV with very low hysteresis, indicating mesoporous materials with very low ordered or non-ordered porosity. There are no evident differences between the two isotherms, neither in shape nor in the maximum adsorbed volume.

The specific surface area is similar in value for both samples, with a slow increase for the Fe_3_O_4_ containing one (Table 1). Total pore volume is also very similar for both samples. The influence of the presence of Fe_3_O_4_ in the composite materials is evident instead on the pore size distribution (Figure 4b). Both materials present very large pore size distributions, ranging from 4 to 100 nm, indicating a multicomponent material with no ordered pore size. The Bch-HCl/MnO_2_ sample shows a maximum at 4 nm and a large plateau with a second maximum at around 100 nm. The large extra peak situated at 20 nm in the Bch-HCl/Fe_3_O_4_/MnO_2_ plot can be attributed to the Fe_3_O_4_ phase of the composite material.

#### 3.1.3. FTIR Analysis

**Figure 5:**
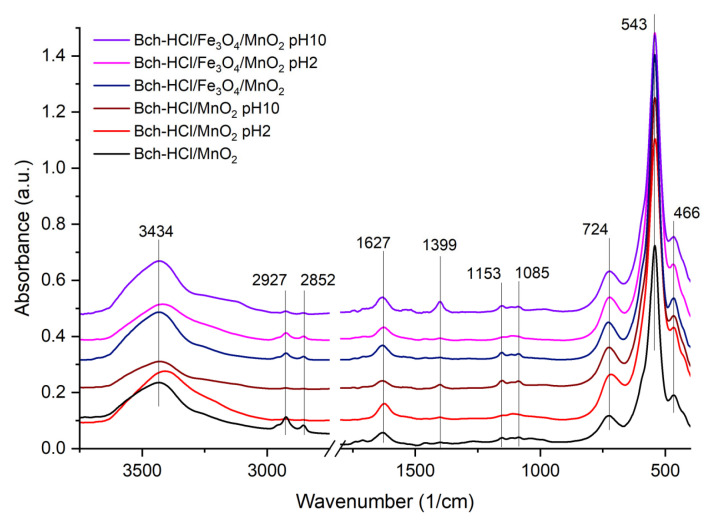
FTIR spectra of Bch-HCl/MnO_2_ and Bch-HCl/Fe_3_O_4_/MnO_2_ samples compared to spectra of these samples exposed to acidic (pH 2) and basic (pH 10) conditions, 3750–400 cm^−1^ spectral domain (spectral break between 2750–1800 cm^−1^).

By analyzing the infrared spectra (Figure 5) of the Bch-HCl/MnO_2_ and Bch-HCl/Fe_3_O_4_/MnO_2_ samples, the following spectral bands can be identified: the band at 3434 cm^−1^ can be associated with the stretching vibration of –OH groups, linked through hydrogen bonds, originating from water adsorbed on the surface of the analyzed material; vibrational bands appear at 2924 and 2854 cm^−1^ due to the symmetric and asymmetric stretching vibrations of C–H bonds in CH_2_ and CH_3_ groups [37].

By comparing the absorption bands in the spectral range 1800–400 cm^−1^ [37], some differences can be identified in the spectra of the two analyzed samples, manifested as minor shifts or changes in intensity of certain spectral bands. These differences are listed in Table 2.

Through the comparison of the FTIR spectra of these samples exposed to acidic (pH 2) and basic (pH 10) environments, no discernible changes were detected, which demonstrates the chemical stability of these materials under altered pH conditions.

#### 3.1.4. XRD Analysis

**Figure 6:**
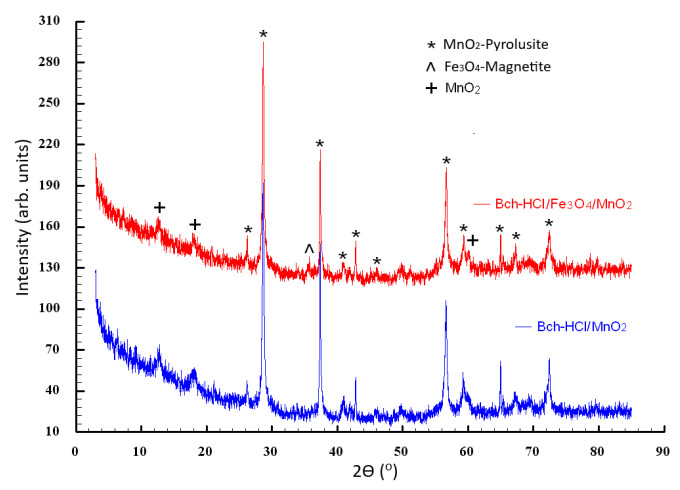
X-ray diffractograms corresponding to the obtained materials.

The X-ray powder diffractograms of the analyzed samples are illustrated comparatively in Figure 6.

These are structurally similar, being characterized by the same diffraction maxima. Thus, two distinct phases of manganese oxide were identified. The first phase, the dominant one, is represented by the tetragonal lattice of manganese dioxide in the form of MnO_2_-Pyrolusite with lattice constants a = 4.38 Å, c = 2.85 Å, space group P4_2_/mmm, which is characterized by a series of peaks appearing at 2θ = 28.7°, 37.3°, 40.8°, 42.7°, 46.2°, 56.6°, 59.3°, 64.8°, 67.2°, and 72.3°. The two diffraction peaks that appear at smaller diffraction angles 2θ = 12.7° 18.1° and the peak at 60.1° also correspond to manganese oxide MnO_2,_ but are found in a polymorphic form, which is also tetragonal but characterized by higher lattice constants, with lattice parameters a = 9.78 Å, c = 2.86 Å, space group I4/m. Additionally, in the case of the Bch-HCl/Fe_3_O_4_/MnO_2_ sample, the presence of iron oxide in the form of magnetite, Fe_3_O_4_, is observed, which is manifested by the existence of the diffraction maximum at 2θ = 35.8°.

### 3.2. Adsorption Process Optimization

#### 3.2.1. Effect of pH and Point of Zero Charge

**Figure 7:**
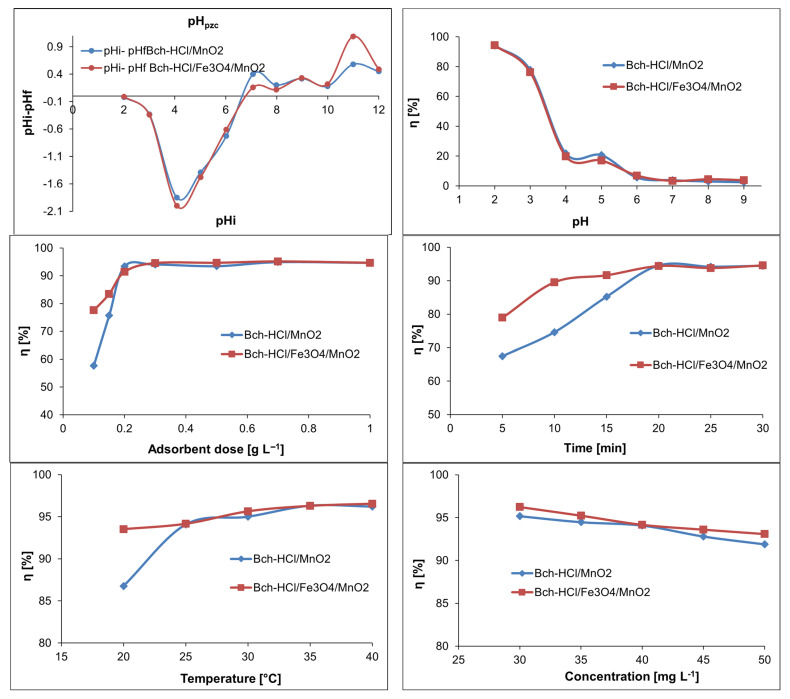
pH_pzc_ equal to the pH point of zero charge, and the effect of variables on paracetamol adsorption on prepared composites.

The point of zero charge (pH_pzc_) is a key parameter in the adsorption process, indicating the nature of active sites on the adsorbent [40]. When the solution’s pH is below the pH_pzc_, the adsorbent surface carries a positive charge, which enhances the adsorption of anionic pollutants. Conversely, when the solution pH exceeds the pH_pzc_, the adsorbent surface becomes negatively charged, favoring the adsorption of cationic contaminants [41]. The influence of pH was investigated by adjusting the drug solution’s pH between 2 and 9 at 25 °C over a period of 20 min. The paracetamol concentration was maintained at 40 mg L^−1^, with an adsorbent dosage of 1 g L^−1^. Figure 7 illustrates the impact of pH on the percentage removal of paracetamol by the prepared composites.

The removal of paracetamol was maximum at pH 2 for both adsorbents and decreased with the increase in the initial pH of the drug solution. This showed that the availability of positively charged groups at the adsorbent surface facilitates the adsorption of paracetamol. The pH 2 of the paracetamol solution at which the pollutant is best adsorbed is lower than the pH_pzc_ (6.64 for Bch-HCl/MnO_2_ and 6.78 for Bch-HCl/Fe_3_O_4_/MnO_2_) of the adsorbent (Figure 7), indicating that the prepared composites remain in their protonated form with a net positive charge on the surface. Consequently, in the future experiments, the pH value of the paracetamol solution was 2.

As reported by Elbagerma and colleagues [42], paracetamol exists as uncharged within the studied solution pH values ranging from 2.0 to 10. Thus, according to the literature, electrostatic attraction was excluded [43]. Also, according to the literature, the removal of polar aromatic pollutants, such as paracetamol, by porous carbonaceous materials involves van der Waals force, hydrogen bonding formation, n-π interaction, and pore-filling [43].

#### 3.2.2. Effect of the Adsorbent Dose

The impact of the adsorbent dose was examined by varying the concentration of the prepared adsorbents from 0.1 to 1 g L^−1^, while maintaining the paracetamol concentration at 40 mg L^−1^ (pH 2) at 25 °C for 20 min.

The removal degree of paracetamol (Figure 7) enhanced with an increasing quantity of adsorbent and it reached a plateau (94.06% for Bch-HCl/MnO_2_ and 94.56% for Bch-HCl/Fe_3_O_4_/MnO_2_) at 0.3 g L^−1^. Above the 0.3 g L^−1^ adsorbent dose, the drug removal percentage shows no significant improvement, probably due to the aggregation of particles that can occur at high adsorbent doses and reduce the accessible surface area [44,45]. Thus, the 0.3 g L^−1^ adsorbent dose was considered optimum and used in subsequent experiments.

#### 3.2.3. Effect of Contact Time

The influence of contact time was evaluated over a range of 5 to 30 min, while maintaining constant conditions: paracetamol concentration at 40 mg L^−1^, pH at 2, and temperature at 25 °C.

The effect of time on the paracetamol removal degree is presented in Figure 7. As the adsorption time increases, the removal rate increases rapidly up to 20 min, after which no change is observed. This behavior is attributed to the availability of active adsorption sites; over time, these sites become occupied, and at extended contact durations, no significant additional adsorption takes place [46]. Accordingly, a 20-minute contact time was considered optimal and was further used for adsorption.

#### 3.2.4. Effect of Temperature

The influence of temperature on paracetamol adsorption on the two synthesized adsorbents was assessed by varying the temperature from 20 °C to 40 °C, while keeping the paracetamol concentration (40 mg L^−1^), solution pH (2), and adsorbent dose (0.3 g L^−1^) constant throughout the experiments.

The influence of the temperature on the removal degree of paracetamol is shown in Figure 7. The results showed that with increasing temperature, the removal degree of paracetamol increases slightly for both adsorbents. The increase in removal rate with temperature may be due to the activation of new sites from the adsorbent surface [47]. After 25 °C, the degree of elimination increases insignificantly. Therefore, 25 °C was considered the optimum temperature for the adsorption of paracetamol on the prepared materials.

#### 3.2.5. Effect of the Initial Drug Concentration

The influence of the initial drug concentration was investigated at 25 °C for 20 min by varying the paracetamol concentration from 30 to 50 mg L^−1^, while maintaining a constant adsorbent dosage of 0.3 g L^−1^. The pH of the paracetamol solutions was maintained at 2. The removal efficiency depends on the change in initial concentration, varying, as shown in Figure 7, from 95 to 91% for Bch-HCl/MnO_2_ and 96 to 93% for Bch-HCl/Fe_3_O_4_/MnO_2_. The decrease in the removal percentage when the drug concentration increases is due to the availability of a smaller quantity of available adsorption sites [48].

### 3.3. Adsorption Isotherm Studies

To determine the adsorption isotherm of paracetamol on the prepared adsorbents, a solution of paracetamol of different initial concentrations (5–40 mg L^−1^) at pH 2 was stirred with a dose of adsorbent of 0.3 g L^−1^, for 20 min at 400 rpm, at different temperatures (20–40 °C).

Isotherm models were applied to analyze the adsorption behavior of paracetamol on the synthesized adsorbents. The Langmuir, Freundlich, and Temkin models, in their linearized forms as described by Lung et al. [36], were utilized, and the corresponding parameters are summarized in Table 3.

**Figure 8:**
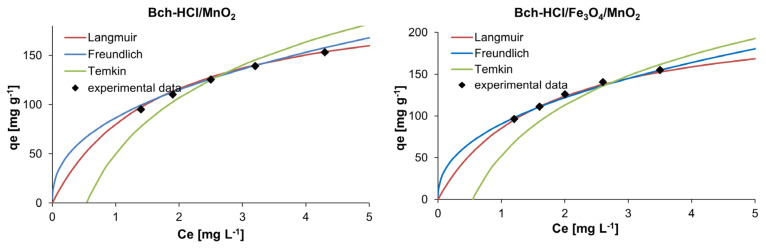
Adsorption behavior of paracetamol on prepared composites at 25 °C, interpreted using Langmuir, Freundlich, and Temkin isotherm models.

The isotherm model validity can be judged by the R^2^ value of each plot. Considering the R^2^ value and the comparison of the isotherms with the experimental data (Figure 8), no model perfectly describes the uptake of paracetamol. However, the Langmuir and the Freundlich isotherms fitted the paracetamol adsorption data best, even though the R^2^ values of the Temkin model were close to them. According to this model, the adsorption of paracetamol takes place on a surface characterized by heterogeneity and multilayer adsorption [41]. Also, the adsorption of paracetamol is favorable under experimental conditions because the values of n are between 0 and 10 [49].

A similar result was reported for paracetamol removal from synthetic wastewater onto biochar derived from solid cypress cones by Akacha et al. [50]. Akpomie et al. have biosynthesized copper oxide nanoparticles, which were used for the adsorption of paracetamol onto their surface. The findings indicate that the Freundlich model fits the adsorption experimental data better [51]. Alakayleh investigated the feasibility of guava leaves’ sulfuric acid-activated carbon for the removal of paracetamol [52]. Various isotherm models were applied to fit the equilibrium data for paracetamol adsorption onto guava leaves’ sulfuric acid-activated carbon. The analysis revealed that the Freundlich model best represented the experimental results. Similarly, the Freundlich isotherm provided the most accurate fit for equilibrium data regarding paracetamol removal from water using activated carbon derived from paper waste and avocado seeds. [53]. Skwarczynska-Wojsa et al. evaluated commercially available granulated activated carbon for effective paracetamol removal from aqueous solutions. Analysis of the equilibrium data indicated that the Freundlich isotherm model most accurately described the adsorption behavior [54]. Sajid et al. studied the transformation of Cannabis sativa (hemp) into activated carbon for paracetamol removal from water. They evaluated both Langmuir and Freundlich adsorption isotherms and found that the Freundlich model provided the best fit to the experimental data [55].

### 3.4. Kinetic Studies

The duration of contact between the adsorbent and paracetamol plays a vital role in understanding the adsorption mechanisms. Kinetic models are used to calculate the key rate constants governing the adsorption process [56]. The adsorption kinetics were analyzed using pseudo-first-order, pseudo-second-order, and intraparticle diffusion models, with the corresponding equations provided by Lung et al. [36]. The determined kinetic parameters are listed in Table 4.

Based on the experiments and calculations performed, the adsorption of paracetamol on Bch-HCl/MnO_2_ and Bch-HCl/Fe_3_O_4_/MnO_2_ was best described by second-order kinetics. In the case of this model, the highest R^2^ values were obtained. Nonetheless, fitting the model alone does not conclusively confirm this mechanism [57].

**Figure 9:**
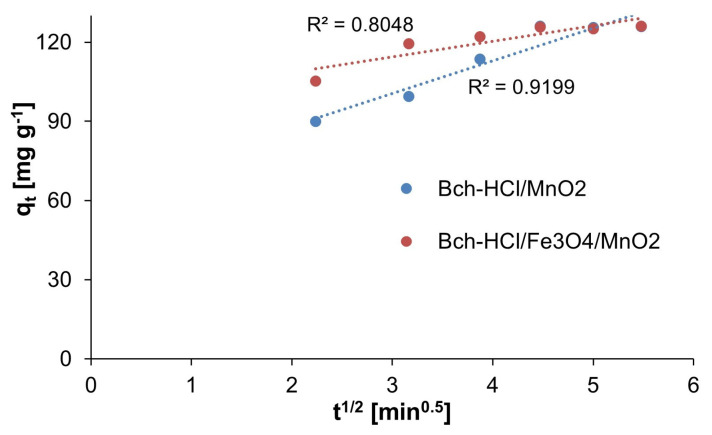
Intraparticle diffusion adsorption kinetics of paracetamol onto Bch-HCl/MnO_2_ and Bch-HCl/Fe_3_O_4_/MnO_2_.

The kinetic data were also analyzed by means of the intraparticle diffusion model to identify the molecular diffusion mechanism. Even if the q_t_ versus t^1/2^ graph shows a linear regression (Figure 9), it fails to pass through the origin. Therefore, the intraparticle diffusion model is not valid, suggesting that paracetamol adsorption was not entirely controlled by intraparticle diffusion [58].

The boundary layer thickness constant values (C = 63.36 mg g^−1^ and 96.64 mg g^−1^, respectively) indicate a significant boundary layer effect. This suggests that external mass transfer from the solution is hindered, while internal mass transfer within the adsorbent is enhanced [50].

Other studies in which the second-order kinetic model was the most suitable model for the removal of paracetamol were when it was used as an adsorbent biochar from cypress cones solid waste [50], biochar from pepper stem [59], and biochar from *Sargassum cymosum macroalgae* [60].

### 3.5. Thermodynamic Parameters

Adsorption thermodynamics differs between systems, and its analysis provides information on the process spontaneity, exothermic/endothermic nature, and the physical or chemical nature of the adsorption [51].

The thermodynamic parameters such as enthalpy variation (ΔH°), entropy variation (ΔS°), and free energy variation (ΔG°) have been calculated using the following equations [61]:

(3) ∆G°=∆H°−T∆S°

(4) $$∆G{}^{\circ}=-RTlnK_{e}^{0}$$

(5) $$lnK_{e}^{0}=\frac{∆S{}^{\circ}}{R}-\frac{∆H{}^{\circ}}{RT}$$

(6) $$K_{e}^{0}=\frac{\left(1000\times K_{L}\times molecular\;weight\;of\;adsorbate\right)\times [Adsorbate]{}^{\circ}}{\gamma }$$

γ-coefficient of activity (dimensionless), [Adsorbate]°—standard concentration of the adsorbate (1 mol L^−1^), and $K_{e}^{0}$—thermodynamic equilibrium constant that is dimensionless.

The thermodynamic parameters values that revealed the behaviors for adsorption of paracetamol on prepared adsorbents are presented in Table 5.

The enthalpy’s negative value confirms the exothermic nature of the adsorption process on both materials. Its values being small and negative suggest that the adsorption has a physical character, involving weak attractive forces [62]. The entropy was positive, which indicates a rise in disorder at the paracetamol/Bch-HCl/MnO_2_ and paracetamol/Bch-HCl/Fe_3_O_4_/MnO_2_ interfaces [50]. Under the experimental conditions, ΔG° values are negative (Table 5), suggesting that the adsorption phenomenon was spontaneous and feasible in the temperature range examined [53]. By analyzing the results obtained, it was observed that the ΔG° values experienced a reduction as the temperature was elevated, suggesting that the process is probably governed by physical adsorption [63]. Similar results were also found for paracetamol removal on Biochar with spherical morphology synthesized from pure glucose and non-spherical biochar obtained from pomelo peel residues [43].

### 3.6. Reusability of the Prepared Composites

**Figure 10:**
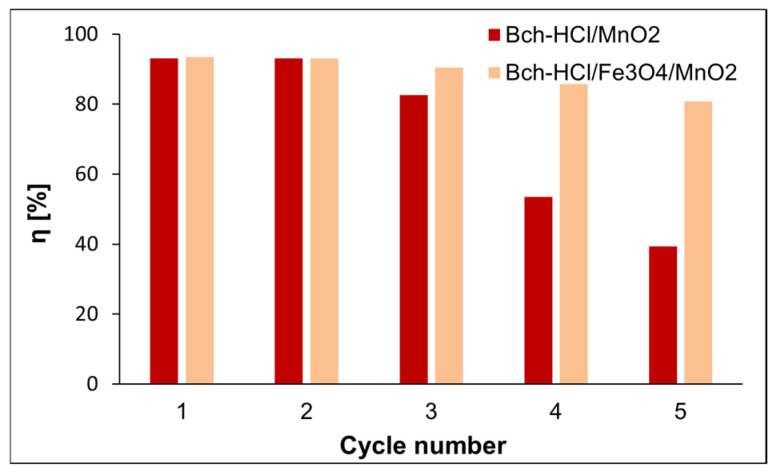
Adsorption of paracetamol by prepared composites in five consecutive cycles.

The reusability of the prepared composites was demonstrated by adsorption–desorption experiments of up to five cycles (Figure 10).

It was observed that the degree of paracetamol removal on the two adsorbents was slightly reduced after each cycle in the case of Bch-HCl/Fe_3_O_4_/MnO_2_, and in the case of Bch-HCl/MnO_2_, it was significantly reduced after the third cycle. Therefore, the Bch-HCl/Fe_3_O_4_/MnO_2_ composite can be used in a series of treatment cycles.

### 3.7. Performance Evaluation

To determine the performance of the prepared composites, their maximum adsorption capacities were compared with those of other materials obtained from agricultural wastes, used for paracetamol removal (Table 6). It was observed that the maximum adsorption potential of biochar obtained from tomato waste and functionalized with metal oxides (Fe_3_O_4_, MnO_2_), obtained in this research, is substantial compared to other adsorbents obtained from agricultural waste. Consequently, Bch-HCl/MnO_2_ and Bch-HCl/Fe_3_O_4_/MnO_2_ can serve as promising materials for efficiently removing paracetamol from water.

## 4. Conclusions

This study demonstrates the efficiency of biochar obtained from tomato waste, functionalized with Fe_3_O_4_ and MnO_2_ nanoparticles, as an economical and environmentally friendly adsorbent for the effective removal of paracetamol from water. The synthesized composites, Bch-HCl/MnO_2_ and Bch-HCl/Fe_3_O_4_/MnO_2_, were characterized and exhibited porous structures with wide pore size distributions, high surface areas, and favorable surface functionalities, as evidenced by BET and FTIR analyses. Both materials demonstrated excellent adsorption performance, with removal efficiencies exceeding 94% under the following optimal conditions: 30 mg L^−1^ paracetamol concentration, pH 2, 0.3 g L^−1^ adsorbent dosage, 25 °C, and 20 min contact time. The influence of Fe_3_O_4_ was evident in the altered pore size distribution and slightly increased surface area, which contributed to enhanced adsorption capacity.

The adsorption behavior was strongly influenced by the pH of the solution, with the highest efficiency observed under acidic conditions. This is attributed to the enhanced positive charge on the adsorbent surface at low pH, which promotes stronger interaction with paracetamol molecules. Analysis of the equilibrium data showed that the Freundlich isotherm provided the best fit, indicating multilayer adsorption on a heterogeneous surface. Kinetic evaluation demonstrated that the process followed a pseudo-second-order model, suggesting that the rate-limiting step may involve chemisorption. Thermodynamic parameters supported the spontaneous nature of the adsorption, as evidenced by negative Gibbs free energy (ΔG°) values. Additionally, the slightly negative enthalpy change (ΔH°) confirmed the exothermic character of the process and suggested that physical adsorption was predominant. The positive entropy change (ΔS°) pointed to increased disorder at the solid–liquid interface during adsorption. Comparison with other biochar-based adsorbents from agricultural waste highlighted the high competitiveness of the tomato waste-derived composites in terms of adsorption capacity and operational efficiency. Additionally, the prepared materials showed fast equilibrium times, high efficiency at low contact times and moderate temperatures, and minimal performance loss across a range of initial paracetamol concentrations.

Overall, this study demonstrates the potential of Fe_3_O_4_- and MnO_2_-modified tomato waste biochar as a sustainable and efficient material designed for the adsorption-based removal of pharmaceutical compounds from water. These findings not only contribute to sustainable waste management by valorizing agricultural by-products but also offer a promising tool to improve water treatment technologies.

## Figures and Tables

**Table 1:** Measured values of specific surface area (S_BET_), average pore diameter (D_m_), and pore volume (V_p_) for the synthesized materials.

Sample	S_BET_ (m^2^ g^−1^)	D_m_ (nm)	V_p_ (cm^3^ g^−1^)
Bch-HCl/MnO_2_	22.6	35.6	0.16
Bch-HCl/Fe_3_O_4_/MnO_2_	26.7	34.5	0.17

**Table 2:** Spectral differences observed between the samples Bch-HCl/MnO_2_ and Bch-HCl/Fe_3_O_4_/MnO_2_ in the 1800–400 cm^−1^ domain.

Bch-HCl/MnO_2_ (cm^−1^)	Bch-HCl/Fe_3_O_4_/MnO_2_ (cm^−1^)	Assignments
1710	1706	stretching vibration of -C=O and amidic N-H
1627	1627	high intensity band of stretching H-O-H, aromatic -C=C- groups
1458	1461	bending vibration of -O-H bonds stretching vibration of -C-OH
1399	1399	
1310 sh	1310	
1266	1285	
1153	1153	
1115	1115	
1085	1085	
1040	1028 sh	stretching vibration of -C-O
991	988	
724	724	stretching vibration of Fe-O (from Fe_2_O_3_)
543	542	sharp, high-intensity band of stretching vibration of Fe-O [38] (from Fe_3_O_4_) and Mn-O (from MnO_2_) [39]
466	466	stretching vibration of Mn-O [39]

**Table 3:** Parameters of adsorption isotherms for paracetamol removal using the prepared composites under different thermal conditions.

	20 °C	25 °C	30 °C	35 °C	40 °C
**Bch-HCl/MnO** **_2_**					
*Langmuir*					
q_m_ [mg g^−1^]	149.25	212.77	232.56	256.41	263.16
K_L_ [L mg^−1^]	0.71	0.60	0.59	0.61	0.61
R^2^	0.9903	0.9962	0.9927	0.9958	0.9950
*Freundlich*					
K_F_ [Lⁿ mg^1−^ⁿ g^−1^]	81.17	86.64	90.01	98.15	99.93
n	4.71	2.43	2.17	1.98	1.76
R^2^	0.9695	0.9995	0.9714	0.9865	0.9909
*Temkin*					
K_T_ [L mg^−1^]	1.36	1.85	1.94	1.93	2.06
b_T_ [J mol^−1^]	31.68	30.25	29.27	26.51	26.33
R^2^	0.9513	0.9962	0.9851	0.9948	0.9964
**Bch-HCl/Fe** **_3_** **O** **_4_** **/MnO** **_2_**					
*Langmuir*					
q_m_ [mg g^−1^]	192.31	222.22	238.10	285.71	294.10
K_L_ [L mg^−1^]	0.75	0.63	0.59	0.47	0.47
R^2^	0.9988	0.9884	0.9822	0.9700	0.9904
*Freundlich*					
K_F_ [L mg^−1^]	89.35	90.41	92.32	94.04	95.57
n	2.87	2.33	2.17	1.81	1.74
R^2^	0.9965	0.9969	0.9946	0.9900	0.9768
*Temkin*					
K_T_ [L mg^−1^]	1.64	1.83	1.88	2.16	2.18
b_T_ [J mol^−1^]	28.42	28.48	28.15	27.96	27.94
R^2^	0.9996	0.9850	0.9793	0.9770	0.9927

**Table 4:** Kinetic model constants for paracetamol adsorption onto the tested adsorbents.

	Bch-HCl/MnO_2_	Bch-HCl/Fe_3_O_4_/MnO_2_
*Pseudo-first order*		
q_e calc_ [mg g^−1^]	130.517	41.267
k_1_ [min^−1^]	0.213	0.170
R^2^	0.7928	0.8778
*Pseudo-second order*		
q_e calc_ [mg g^−1^]	140.845	131.579
k_2_ [g mg^−1^ min^−1^]	0.002	0.001
h [mg g^−1^ min^−1^]	42.017	123.457
R^2^	0.9953	0.9997
*Intraparticle diffusion*		
K_d_ [mg g^−1^ min^−0.5^]	12.399	5.936
C	63.362	96.644
R^2^	0.9199	0.8048

**Table 5:** Thermodynamic parameters for the adsorption of paracetamol on prepared adsorbents.

Temperature [K]	Bch-HCl/MnO_2_			Bch-HCl/Fe_3_O_4_/MnO_2_		
	ΔH° [J mol^−1^]	ΔS° [J mol^−1^ K^−1^]	ΔG° [kJ mol^−1^]	ΔH° [J mol^−1^]	ΔS° [J mol^−1^ K^−1^]	ΔG° [kJ mol^−1^]
293 298 303 308 313	−0.015	86.782	−25.427 −25.861 −26.295 −26.729 −27.163	−0.004	38.402	−11.252 −11.444 −11.636 −11.828 −11.020

**Table 6:** Comparison of adsorptive capabilities of various agriculturally derived adsorbents for paracetamol removal.

Adsorbent	Adsorption Capacity (mg g^−1^)	Reference
Bch-HCl/MnO_2_	212.77	Present study
Bch-HCl/Fe_3_O_4_/MnO_2_	222.22	Present study
Biochar from cypress cones solid waste	59.86	[50]
Biochar from pomelo peel wastes	147	[43]
Biochar from pepper stem	354.66	[59]
Biochar from *Sargassum cymosum macroalgae*	12.34	[60]
Biochar from microalgae biomass	74.92	[64]
N-dopped biochar	105.60	[64]
Biochar from banana peel	49.43	[65]
BC-Act-COP	298	[65]
BC-Act-IP	237	[66]
Ag_2_O-CuO/biochar	55.25	[67]
Rice hull activated carbon	169.49	[68]
*Cannabis sativum Hemp* activated carbon	16.18	[55]
Sulfuric acid-activated carbon from guava leaves	13.30	[52]
Pine fruit waste activated carbon	41.7	[49]
Activated carbon from spent tea leaves	59.20	[22]
Groundnut shell	3.02	[69]
Fe_3_O_4_/C composite obtained from tamarind shell biomass	8.10	[51]
CNT-COOH/MnO_2_/Fe_3_O_4_ composite	80.64	[36]
CuO nanoparticles	64.52	[70]
ZnO nanoparticles	7.87	[71]
TiO_2_/Fe_2_O_3_/Chitosan	175.43	[72]

## Data Availability

The original contributions presented in this study are included in the article. Further inquiries can be directed to the corresponding author.
